# Possible primary immunodeficiency presenting with gastrointestinal symptoms: Case report and minireview

**DOI:** 10.3892/etm.2013.1178

**Published:** 2013-06-25

**Authors:** HONGGANG WANG, MIN WANG, ZHINING FAN, GUOZHONG JI, FAMING ZHANG

**Affiliations:** Institute of Digestive Endoscopy and Medical Center for Digestive Diseases, Second Affiliated Hospital of Nanjing Medical University, Nanjing, Jiangsu 210011, P.R. China

**Keywords:** primary immunodeficiency, gastrointestinal symptoms, prednisone, follow-up

## Abstract

Primary immunodeficiency is a disease characterized by reduced levels of serum immunoglobulins and multiple clinical manifestations. Patients with primary immunodeficiency frequently present with gastrointestinal symptoms, such as diarrhea, malabsorption and weight loss. The mainstay of treatment is replacement therapy with intravenous immunoglobulin (IVIG). In the current study, we report the case of a 23-year-old man with symptoms of chronic diarrhea, malabsorption and weight loss that had been apparent for two years. Subsequent to being diagnosed with possible primary immunodeficiency, the patient was treated with 30 mg/day oral prednisone for one month. The prednisone was then tapered weekly by 5 mg until withdrawal. Three months later, the patient’s clinical symptoms disappeared and his quality of life improved. During the subsequent nine months follow-up, the patient was able to work without suffering any effects from his illness. The body weight of the patient increased and plasma albumin levels were normal. In conclusion, this study describes the case of a patient with primary immunodeficiency-related gastrointestinal symptoms who responded well to oral prednisone treatment.

## Introduction

Primary immunodeficiencies are disorders in which part of the body’s immune system is missing or does not function correctly. Most primary immunodeficiencies are genetic disorders and the majority are diagnosed in children under the age of one, although milder forms may not be recognized until adulthood (1). The diagnostic criteria for primary immunodeficiencies were established in 1999 and distinguish between ‘definitive’, ‘probable’ and ‘possible’ in the diagnosis of primary immunodeficiency. A ‘definitive’ diagnosis is made when the patient has a >98% chance of the same diagnosis being made in the following 20 years; this level of diagnosis is achievable with the detection of a genetic mutation or very specific circumstantial abnormalities. A ‘probable’ diagnosis is made when a genetic diagnosis is not applicable, although the patient has all the other characteristics of a particular disease; the chance of the same diagnosis being made 20 years later is estimated to be 85–97%. A ‘possible’ diagnosis is made when the patient exhibits some of the characteristics of a disease, but not all (2). The treatment of primary immunodeficiency depends foremost on the nature of the abnormality. This may range from immunoglobulin replacement therapy in antibody deficiencies, in the form of intravenous immunoglobulin (IVIG), to hematopoietic stem cell transplantation for severe immunodeficiency (3). In the current study, we describe the case of a successful diagnosis of primary immunodeficiency and the treatment of the patient with prednisone.

## Case report

A 23-year-old male was admitted to the Second Affiliated Hospital of Nanjing Medical University (Nanjing, China), complaining of chronic diarrhea. The patient had suffered from chronic diarrhea without blood or mucus for almost two years. The diarrhea took the form of unshaped, liquid stools and occurred two to four times a day. The patient had lost ∼8 kg in weight, despite having a healthy appetite, and had received long-term treatment in other university hospitals, based on the diagnosis of protein-losing gastroenteropathy. Following the transferral of the patient to the Second Affiliated Hospital of Nanjing Medical University, apparent edema was observed in the lower limbs, in addition to a medium volume of ascites. Routine laboratory tests revealed that the counts of white blood cells (including accurate lymphocyte and granulocyte counts), hemoglobin and platelets were generally normal. The erythrocyte sedimentation rate was 21 mm/h and the C-reactive protein level was 1 mg/l. The values for liver enzymes, urea nitrogen, creatinine, glucose and electrolytes were normal. The total protein level in the serum was 33 g/l, with 15 g/l albumin and 18 g/l globulin. Thyroid function [reverse triiodothyronine (rT3), reverse tetraiodothyronine (rT4), total T3 (TT3), total T4 (TT4) and thyroid-stimulating hormone (TSH)] and coagulation tests were normal and the standard autoantibody screen [antinuclear antibodies (ANA), anti-double-stranded DNA (anti-dsDNA) and antineutrophil cytoplasmic antibodies (ANCA)] was negative. The levels of immunoglobulin (Ig) G, IgM and IgA were 7.32, 0.28 and 1.55 g/l, respectively, and urinalysis and 24 h-urine protein measurements were normal. The microbiological test results excluded certain viral infections (hepatitis B and C and HIV), while repeated stool cultures, parasites and ova, and occult blood in the stool were all normal. The size of the liver was normal, while the spleen appeared marginally enlarged when examined using ultrasound. A chest radiograph and abdomino-pelvic computed tomography (CT) scan did not reveal anything of note. In order to investigate the cause of the gastrointestinal symptoms, an upper gastrointestinal endoscopy, colonoscopy and double-balloon enteroscopy were performed. Biopsy specimens revealed villus blunting and flattening/atrophy in the duodenum, while nodular lymphoid hyperplasia and villus blunting and flattening/atrophy were observed in the ileum.

**Figure 1.**
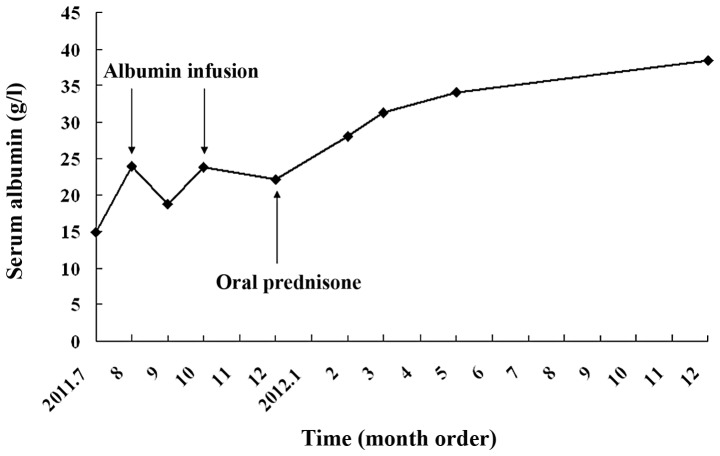
The serum albumin level increased significantly following prednisone treatment. The reference value of albumin is 35.0–55.0 g/l.

**Figure 2.**
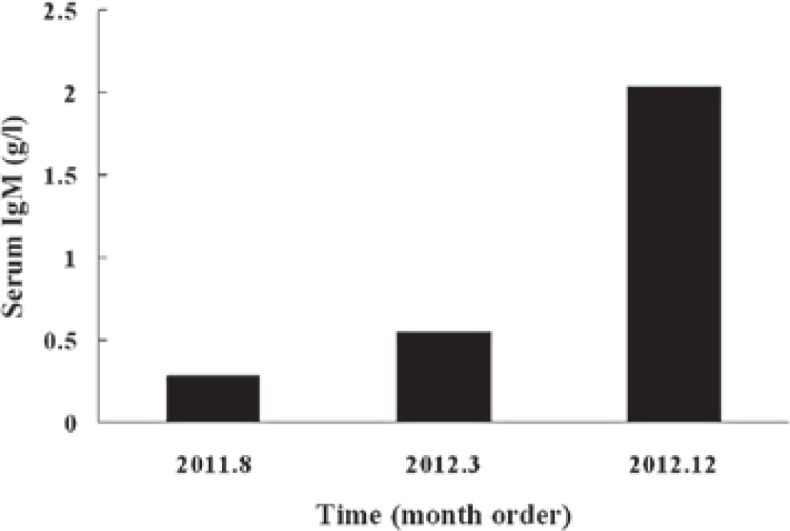
The immunoglobulin (Ig) M level increased with prednisone treatment. The reference value of IgM is 1.0–3.0 g/l.

According to these clinical and laboratory findings, the patient was diagnosed with possible primary immunodeficiency due to IgM deficiency. Considering the patient’s low serum albumin level, albumin infusion therapy was prescribed. Following this, it was decided to initiate treatment with 30 mg/day oral prednisone for one month, prior to weekly tapering by 5 mg until withdrawal. This led to a progressive clinical improvement. In addition, the patient was supplied with calcium, zinc and vitamin supplements. Three months later, the patient was producing 1 or 2 stools/day, had gained ∼10 kg in body weight and was not observed to have any edema of the limbs. During the subsequent nine months follow-up, the patient was able to work without suffering any effects from the illness. A significant increase in the patient’s serum albumin level was observed following the prednisone treatment (Fig. 1) and the serum IgM level also returned to normal (Fig. 2). This study was approved by the Ethics Committee of Nanjing Medical University. Written informed consent was obtained from the patient.

## Discussion

A survey of 10,000 American households revealed that the prevalence of diagnosed primary immunodeficiency approaches 1:1,200 (4). This figure does not take into account individuals with mild immune system defects who have not received a formal diagnosis. Milder forms of primary immunodeficiency, such as selective IgA deficiency, are fairly common, with random groups of individuals (such as otherwise healthy blood donors) exhibiting a rate of 1:600. Other disorders are distinctly more uncommon, with incidences between 1:100,000 and 1:2,000,000 (1). Since the diseases are particularly rare in Asia, the incidence rate in Asia is much lower than that in America and Europe. There may be a requirement for gastroenterologists in Asian countries to obtain a greater recognition of this disease.

IgM deficiency, which is not very frequently encountered, may be classified as one of the primary immunodeficiency diseases. The prevalence of gastrointestinal disorders in patients with primary immunodeficiency diseases ranges from 5 to 50% (5,6). Gastrointestinal disorders (particularly diarrhea and malabsorption) may be the most prominent or the sole complaints in certain patients (7,8). Common variable immunodeficiency (CVID) is the most frequently-encountered primary immunodeficiency (9). The IgG concentrations in CVID are at least two standard deviations below the mean for the age of the patient and, in the majority of cases, are accompanied by low concentrations or an absence of IgA, IgM or the two combined. Patients with CVID typically present with atypical inflammatory gastrointestinal disease, resulting in diarrhea, malabsorption and weight loss (10). Nodular lymphoid hyperplasia and loss of villi are frequently observed in the biopsies of patients with CVID. It has been revealed that up to 60% of patients with untreated CVID develop diarrhea, with 10% developing idiopathic malabsorption with weight loss (11). The mainstay treatment is replacement therapy with IVIG (12–15), and this reduces the number of bacterial infections (16) and most likely enhances survival (17). However, the immunoglobulin therapy has little effect on gastrointestinal symptoms.

In the present case, diarrhea, malabsorption and weight loss were the main clinical manifestations. Furthermore, the patient’s serum albumin level was too low, resulting in edema in the lower limbs. The absorption of albumin may be associated with villus blunting and flattening/atrophy in the small intestine. According to the clinical features and laboratory tests, the patient was diagnosed with primary immunodeficiency, a CVID-like disorder, due to the presence of low IgM with normal IgG levels. Thus, selective IgM deficiency was apparent in this case, for which the common treatment is IVIG. However, IVIG is reported to have little effect on gastrointestinal symptoms (7). Despite the fact that the patient had normal IgG levels and was undergoing intravenous replacement therapy every 3 weeks, the diarrhea continued (18). It has been demonstrated that corticosteroid therapy improves diarrhea in patients with CVID through the inhibition of the immune inflammatory response (8). In the present case, oral prednisone was used. The initial dose was 30 mg/day for one month, and this was then tapered weekly by 5 mg until withdrawal. Following the prednisone therapy, the patient’s symptoms dramatically improved. However, it must be noted that it was necessary to consider and treat the potential side-effects during long-term treatment with corticosteroids. The further follow-up confirmed the diagnosis and the success of the treatments.

Although the majority of patients with primary immunodeficiency are able to obtain a good efficacy in the short-term following treatment, relapse is common. Further investigations into the etiology of the disease and novel treatments are required. Interestingly, the recent emerging application of fecal microbiota transplantation has shown its promising value as a simple treatment for primary immunodeficiency with intestinal symptoms and other refractory intestinal diseases, since previous studies highlighted these aspects (19,20). However, further clinical studies with more samples are required.

In conclusion, the diagnosis, treatment and follow-up of the present case provided increased clinical experience and recognition of patients with primary immunodeficiency, particularly in patients from Asia.
